# DECT-CLUST: Dual-Energy CT Image Clustering and Application to Head and Neck Squamous Cell Carcinoma Segmentation

**DOI:** 10.3390/diagnostics12123072

**Published:** 2022-12-06

**Authors:** Faicel Chamroukhi, Segolene Brivet, Peter Savadjiev, Mark Coates, Reza Forghani

**Affiliations:** 1 IRT SystemX, 2 Boulevard Thomas Gobert, 91120 Palaiseau, France; 2 Electrical and Computer Engineering Department, McGill University, Montreal, QC H3A 0G4, Canada; 3 Augmented Intelligence and Precision Health Laboratory (AIPHL), Department of Radiology, McGill University, Montreal, QC H3G 1A4, Canada; 4 Radiomics and Augmented Intelligence Laboratory (RAIL), Department of Radiology and the Norman Fixel Institute for Neurological Diseases, University of Florida College of Medicine, Gainesville, FL 32610, USA

**Keywords:** spectral image clustering, dual-energy CT imaging, mixture models, functional data analysis, HNSCC cancer

## Abstract

Dual-energy computed tomography (DECT) is an advanced CT computed tomography scanning technique enabling material characterization not possible with conventional CT scans. It allows the reconstruction of energy decay curves at each 3D image voxel, representing varied image attenuation at different effective scanning energy levels. In this paper, we develop novel unsupervised learning techniques based on mixture models and functional data analysis models to the clustering of DECT images. We design functional mixture models that integrate spatial image context in mixture weights, with mixture component densities being constructed upon the DECT energy decay curves as functional observations. We develop dedicated expectation–maximization algorithms for the maximum likelihood estimation of the model parameters. To our knowledge, this is the first article to develop statistical functional data analysis and model-based clustering techniques to take advantage of the full spectral information provided by DECT. We evaluate the application of DECT to head and neck squamous cell carcinoma. Current image-based evaluation of these tumors in clinical practice is largely qualitative, based on a visual assessment of tumor anatomic extent and basic one- or two-dimensional tumor size measurements. We evaluate our methods on 91 head and neck cancer DECT scans and compare our unsupervised clustering results to tumor contours traced manually by radiologists, as well as to several baseline algorithms. Given the inter-rater variability even among experts at delineating head and neck tumors, and given the potential importance of tissue reactions surrounding the tumor itself, our proposed methodology has the potential to add value in downstream machine learning applications for clinical outcome prediction based on DECT data in head and neck cancer.

## 1. Introduction

Computed tomography (CT) has been one of the most common and widespread imaging techniques used in the clinic for the last few decades. There is increasing interest in a more advanced CT technique known as dual-energy CT (DECT) or spectral CT that enables additional material or tissue characterization beyond what is possible with conventional CT. In conventional CT, X-rays are emitted at a certain level of energy, whereas in DECT, they are emitted at two separate energy levels, which brings important benefits as compared to standard CT. First, since different materials can have different attenuation coefficients at different energy levels, DECT allows for the separation of materials with different atomic numbers. In particular, DECT enables the computation of image attenuation levels at multiple effective energy levels. This results in the association of a decay curve with each reconstructed image voxel, representing energy-dependent changes in attenuation at that body location. Conventional CT imaging is the first-line modality for the clinical evaluation of many different types of known or suspected cancers in adults [1]. However, because of the properties described above, there has been an increased interest in the use of DECT in oncology in recent years, as it provides a new and exciting way of characterizing tumors as well as their surrounding tissues.

Current expert evaluation of CT scans of head and neck cancer patients in clinical practice is largely based on qualitative image evaluation for the delineation of the tumor anatomic extent and basic two- or three-dimensional measurements. However, an increasing body of evidence suggests that quantitative texture or radiomic features extracted from CT images can be used to enhance diagnostic evaluation, including the prediction of tumor molecular phenotypes, prediction of therapy response, and outcome prediction [2,3,4,5]. The typical radiomic approach for tumor evaluation can be separated into two steps: (i) identification and segmentation of the tumor in the image; and (ii) prediction of a clinical endpoint of interest based on features extracted from the segmented image region. As such, the ability of the segmentation algorithm to correctly target the tumor region is the first essential step in this process. If performed by an expert, manual processing is prohibitively time-consuming and prone to intra- and inter-observer variability. This step is ideally suited for computerized analysis to make these types of analyses feasible and more reproducible. When conventional single-energy CT (SECT) scans represent a 3D image of a patient, DECT scans may be viewed as a 4D image: a 3D body volume over a range of spectral attenuation levels. The latter dimension provides, for each voxel, a decay curve representing energy-dependent changes in attenuation, enabling tissue characterization beyond what is possible with conventional CT [6,7].

DECT has been shown to improve qualitative image interpretation for the evaluation of head and neck cancer and preliminary results also suggest that the energy-dependent curves associated with each image voxel can be used to improve predictions using radiomic approaches [8,9,10,11,12]. However, there is currently no widely accepted method for the use of spectral data from DECT scans for radiomic type studies. In this study, we propose a clustering method that incorporates the spectral tissue attenuation curves as a fourth dimension of the 3D representation of tissue voxels in head and neck DECT scans. We demonstrate that by combining spectral information from the voxel-associated curves and spatial information from the voxel coordinates, we can create a segmentation map with high concordance with tumoral tissue voxels. The proposed model provides a clustering of high qualitative aspect, that can act as the basis for identifying tumor or peritumoral regions to be used in subsequent radiomic studies on DECT scans.

In this paper, we evaluate the application of DECT to head and neck squamous cell carcinoma (HNSCC). Current image-based evaluation of HNSCC tumors in clinical practice is largely qualitative, based on a visual assessment of tumor anatomic extent and basic one- or two-dimensional tumor size measurements. However, the frequently complex shape of mucosal head and neck cancers and at times poorly defined boundaries and potential adjacent tissue reactions can result in a high inter-observer variability in defining the extent of the tumor [13,14,15,16], especially among radiologists without sub-specialty expertise in head and neck imaging. Furthermore, there is strong evidence that alterations of gene expression and protein–protein interactions in the peri-tumoral tissue or normal adjacent tissue may play a critical role in the evolution and risk of recurrence of HNSCC tumors (e.g., [17,18]). Yet these adjacent regions may not be obviously salient upon visual image examination. For all these reasons, the accurate and consistent determination of a predictive region around the tumor is essential, both for conventional staging which determines patient management and for future automated quantitative image-based predictive algorithms based on machine learning. Such a predictive region may include the tumor itself, but also surrounding tissues of biological relevance.

With these considerations in mind, DECT provides new and unexplored opportunities to answer an important question: what can be learned from DECT about tumor heterogeneity and its associations with surrounding tissues? As a first step toward answering this question, in this paper, we adapt functional data analysis (FDA) techniques to DECT data in order to explore underlying patterns of association in and around the tumor. FDA is a classical branch of statistics dedicated to the analysis of functional data, in situations where each data object is considered a function. This is particularly appropriate for DECT image data, as each 3D image voxel is associated with a curve of image intensity decay over multiple reconstructed energy levels (more details are provided below in Section 2.1). Thus, we adapt FDA statistical models for the clustering of 3D image voxels based on the full functional information provided by the decay curves associated with each voxel. More specifically, the architecture underpinning our proposed method is a functional mixture model, where the mixture component densities are built upon functional approximation of the spectral decay curves at each image voxel, and the mixture weights are constructed to integrate spatial constraints. We then derive an expectation–maximization (EM) algorithm to the maximum likelihood estimation (MLE) of the model parameters.

To our knowledge, this is the first article to propose spatial clustering utilizing the full spectral information available in DECT data, based on an appropriate FDA statistical framework. Existing methods for automatic tumor delineation in DECT (reviewed in detail in Section 2.2) are mostly based on deep learning techniques and utilize only a small subset of the available information, due to the sheer amount of 4D (spatial + spectral) data available in a single DECT scan.

We apply the proposed methodology on 91 DECT scans of HNSCC tumors, and we compare our results to manually traced tumor contours performed by an experienced expert radiologist. We also compare to other baseline clustering methods. However, tumor segmentation on its own is not a clinical outcome. A full demonstration of the clinical utility of our method necessitates an analysis of its ability to predict actual clinical outcomes, and how this prediction performance compares to the performance in the case of manually drawn contours, or contours drawn using alternative automatic methods. We leave this prediction analysis for a subsequent paper. As the first article to adapt FDA statistical tools to DECT data, the main focus of the present paper is on the statistical methodology and on algorithm development. As such, we can summarize our contributions as follows:

1. We extend the statistical framework of mixture models to the spatio-spectral heterogeneous DECT data. In particular, DECT energy decay curves observed at each image voxel are modeled as spatially distributed functional observations;
2. We develop unsupervised learning algorithms for clustering by incorporating full spectral information from DECT data;
3. To our knowledge, this is the first time that these models are applied to DECT;
4. The source codes of our algorithms are publicly available https://github.com/fchamroukhi/DECT-CLUST (accessed on 28 October 2022), free of charge.

The rest of this paper is organized as follows; First, as a background, we describe in Section 2 related work on dual-energy CT and dedicated segmentation methods. Then, in Section 3, we introduce the proposed methodology and present the developed algorithms. Section 4 is dedicated to the experimental study and the obtained quantitative results are provided in Section 5. Finally, in Section 6, we discuss the proposed approach and the obtained results.

## 2. Background and Related Work

### 2.1. Dual-Energy CT

The use of DECT techniques has very recently attracted interest in different clinical applications, including diagnostics; for example using DECT for the improved detection of portal vein thrombosis via virtual monoenergetic reconstructions [19], for reducing visceral-motion-related artifacts on the liver by comparing different CT scanner techniques [20], or also using DECT of the heart to the study of coronary artery disease [21].

**Figure 1:**
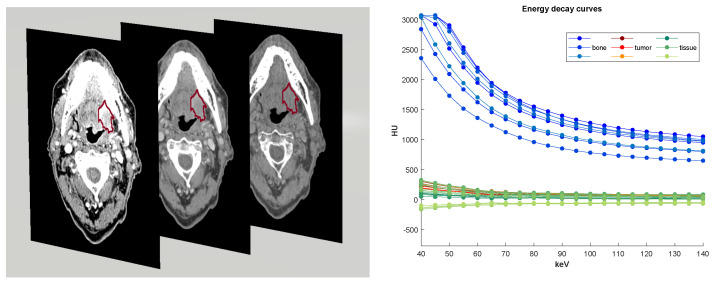
(**Left**) 2D slices of VMIs at 40,65,140 keV with tumor contour in red. At lower energy levels, VMIs are more constrated; at higher levels, VMIs are less noisy. A VMI at 65 keV is similar to a standard CT scan. (**Right**) Examples of decay curves for different body locations. A blue (resp. red, green) curve represents attenuation information stored at one voxel within bone (resp. tumor, tissue).

DECT data may be viewed as a 4D image of a patient: a 3D body volume over a range of energy levels. The dual-energy image acquisition using two X-ray energy peaks at the source provides enough attenuation information to be combined and to be able to reconstruct a curve at multiple “virtual monochromatic" energy levels. These simulate what the attenuation (in Hounsfield units; HU) would be if the study was acquired with a monochromatic X-ray beam at that energy value (in kilo-electron-Volt; keV). The reconstructed curve of attenuation numbers over each energy level translates the energy-dependent changes and is commonly called the spectral Hounsfield unit attenuation curve, or an *energy decay curve* [7]. In our method, we will make use of this spectral information through functional approximations, and thus consider the curves as functional observations. An energy decay curve is calculated for each image voxel, and thus, a DECT scan is represented as a 4D image with 3 dimensions for X, Y and Z spatial coordinates and 1 dimension for energy level coordinates. The virtual monochromatic image (VMI) is the 3D image representation at a given energy level. See Figure 1 (left) for examples of a 2D slice from different VMIs and Figure 1 (right) for examples of decay curves for different tissue characteristics.

### 2.2. Segmentation of Dual-Energy CT Data

Segmentation is a process of delineating an image region of interest. For example, radiation oncologists usually manually segment tumors for radiation planning. Automatic tumor segmentation has a long history of developments: from knowledge-driven early techniques to data-driven newer techniques, algorithms aim to extract image features to make a decision on region boundaries [22]. However, this process remains challenging in medical imaging due to the heterogeneity over the image or the acquisition process; most of the current algorithms need manual adjustments on the result [23].

In head and neck CT imaging, the difficulty to contour precisely a tumor region results in large inter-observer variability in the segmentation results, even among trained radiation oncologists. A study among radiologists from 14 different institutions obtained a median Dice similarity score (DSC) ranging from 0.51 to 0.82 [15], depending on the delineation criteria used. Another study assessing the same variability among 3 experienced radiologists over 10 tumors obtained a mean DSC of 0.57 [16].

To the best of our knowledge, only a few studies have focused on DECT segmentation. These employ deep learning approaches [24,25,26]. The four dimensions of the data required workarounds in order to apply neural networks. For example, using two VMIs sampled from the energy level spectrum, one at a low- and one at a high-energy level, Chen et al. in [24] merged the two VMIs in a layer connected to a U-Net architecture [27]. Wang et al. in [26] learned features from two pyramid networks on the two VMIs independently and combined them through deep attention into a mask-scoring regional convolutional neural network (R-CNN). They achieved good performance in segmenting large-sized organs (DSC larger than 0.8), and performance was less impressive for small-sized organs (DSC between 0.5 and 0.8). Deep learning techniques have indeed revolutionized the world and are very popular in many domains. However, despite what they can provide as good results in practice, we note that as a mixture model-based approach, our proposed approach is not necessarily as complex as a deep learning one can be and is not regarded as a black box. It also enjoys interpretability and relies on the statistical sound background of mixture models and the desirable properties of the EM algorithm, in particular, the fact of monotonically improving the loglikelihood as a loss-function. It is also user-friendly, which can be useful in particular for clinical use, and its implementation is also quite simple.

### 2.3. Decay Curve Clustering via Functional Data Analysis

FDA aims to represent infinite-dimensional functional data into a finite-dimensional vector of coefficients [28]. To achieve this, FDA consists in expanding functional data into function bases. One approach relies on projection on bases which consist in projecting functional data onto finite dimensional function bases (e.g., splines, B-splines, polynomials, Fourier, and wavelet). It associates a finite vector of projection coefficients. This is what we use in this paper. Analogously another common approach would be to run a functional principal component analysis (fPCA) to obtain a basis of eigenfunctions of the covariance of the process describing our functional data. It associates a (truncated) projection vector of PCA coefficients.

Our objective is to partition our data, modeled with FDA, in different groups of voxels having similar decay curve characteristics. Among the available clustering approaches (e.g., centroid-based clustering, such as k-means; connectivity-based clustering, such as hierarchical clustering; density-based clustering, such as DBSCAN [29]; and distribution-based clustering with model-based methods), since we have a model for each decay curve, a model-based approach is preferred.

Model-based clustering is a thoroughly developed field [30,31], particularly for multivariate analysis. Model-based clustering approaches rely on the finite mixture modeling framework [32] to represent the density of a set of independent multivariate observations and on an optimization algorithm to automatically find a partition into groups of such observations.

To represent different groups of data, mixture models assume each datum to follow a known distribution (e.g., Gaussian), and build a mean representation (i.e., model) for each group of data. The mixture model calculates, for each data point, a value defined by the sum over k={1…#groups}, of the probability distribution function (pdf) that this point belongs to group *k* model, emphasized by a weight giving a higher or lower chance of belonging to this group (derived in Section 3.1). Mixture models have the advantage of being interpretable, parametric, thus well-understood, and flexible, as the pdf modeling the data in each cluster can be chosen explicitly.

The expectation–maximization (EM) algorithm [33] is a popular and adapted tool with desirable properties that can be used to conduct an iterative estimation of the mixture model parameters and thus the cluster membership probabilities.

Mixture models for clustering have been applied and adapted to different kind of data, including time-series data [34], gene expression data [35], 3D noisy medical images [36], or spatio-temporal data (non image data) [37]. They also have been recently investigated for functional data [38], and thus provide an avenue to model the spectral decay curves, but in this context of spectral images, we also need to incorporate the spatial information into the clustering.

A related idea was proposed in [39] to develop a spatio-temporal mixture of hidden process models for fMRI analysis. The authors built a temporal probabilistic model, and reshaped the prior probability with spatial constraints to determine a “region of influence” for the temporal model. A specification of our model covers this approach, and our model goes further in generalizing it via the construction of more flexible Gaussian-mixture weights around the spatial coordinates. The resulting model enjoys better numerical learning properties with faster convergence due to closed-form updating rules for the spatial weights parameters. Alternative constructions of the proposed mixture model are also presented in order to validate the method and to accommodate potential user specifications.

## 3. Methodology

### 3.1. Generative Modeling Framework

We adopt the framework of generative modeling for image clustering using different families of extended mixture distributions. The general form of the generative model for the image assumes that the *i*th datum (i.e., pixel and voxel) in the image has the general semi-parametric mixture density:

(1) $$f_{i}\left(\theta \right)=\sum_{k=1}^{K}\pi_{ik}f_{i}\left(\theta_{k}\right)\;,$$

which is a convex combination of *K* component densities, $f_{i}\left(\theta_{k}\right)$, k∈[K]={1,…,K}, weighted by non-negative mixture weights $\pi_{ik}$ that sum to one, that is $\sum_{k=1}^{K}\pi_{ik}=1$ for all *i*, i∈[n]. The unknown parameter vector θ of density (1) is composed of the set of component density parameters $\left\{\theta_{k}\right\}$ and their associated weights $\left\{\pi_{ik}\right\}$, i.e., $\theta ={\left\{\pi_{ik},\theta_{k}\right\}}_{k=1}^{K}$.

From the perspective of model-based clustering of the image, each component density can be associated with a cluster, and hence the clustering problem becomes one of parametric density estimation. Suppose that the image has *K* segments and let $Z_{i}\in \left[K\right]$ be the random variable representing the unknown segment label of the *i*th observation in the image. Suppose that the distribution of the data within each segment k∈[K] is $f(\theta_{k})$, i.e, $f_{iZ_{i}=k}\left(\theta \right)=f_{i}\left(\theta_{k}\right)$. Then, from a generative point of view, model (1) is equivalent to *(i)* sampling a segment label according to the discrete distribution with parameters being the mixture weights $\pi =\{\pi_{1},\ldots ,\pi_{K}\}$, then *(ii)* sampling an observation Imi from the conditional distribution $f(\theta_{k})$. Given a model of the form (1) represented by $\hat{\theta }$, typically fitted by maximum likelihood estimation (MLE) from the *n* observations composing the image Imn, as

(2) θ^∈argmaxθ∈ΘlogL(θImn)

where L(θImn) is the likelihood of θ given the image data Imn, then, the segment labels can be determined via the Bayes’ allocation rule,

(3) Z^i=argmaxk∈[K]P(Zi=kIm(i);θ^),

which consists of maximizing the conditional probabilities

(4) P(Zi=kIm(i);θ^)=π^ikfi(θ^k)fi(θ^)·

that the *i*th observation originates from segment *k*, k∈[K], given the image data and the fitted model.

Model (1) has many different specifications in the literature, depending on the nature of the data generative process, resulting in a multitude of choices for the mixture weights and for the component densities. Mixtures of multivariate distributions [32] are in particular more popular in model-based clustering of vectorial data using multivariate mixtures. These include multivariate Gaussian mixtures [30,31], where $\pi_{ik}=\pi_{k}$, ∀i are constant mixture weights, and the component densities $f_{i}\left(\theta_{k}\right)=\phi_{i}\left(\mu_{k},\Sigma_{k}\right)$ are multivariate Gaussians with means $\mu_{k}$ and covariance matrices $\Sigma_{k}$. Mixtures of regression models, introduced in [40], are common in the modeling and clustering of regression-type data. For example, in the widely used Gaussian regression mixture model [41], we have constant mixing proportions, i.e., $\pi_{ik}=\pi_{k}$, and the mixture components $f_{i}\left(\theta_{k}\right)$’s are Gaussian regressors $\phi (\cdot ,\beta_{k}^{⊤}x_{i},\sigma_{k}^{2})$ with typically linear means $\beta_{k}^{⊤}x_{i}$ and variances $\sigma_{k}^{2}$ in the case of a univariate response.

In this paper, we consider a more flexible mixture of regressions model in which both the mixture weights and the mixture components are covariate-dependent, and are constructed upon flexible semi-parametric functions. More specifically, in this full conditional mixture model, the mixture weights $\pi_{ik}$ are constructed upon parametric functions $\pi_{ik}=\pi_{k}\left(\cdot ,x_{i};\alpha \right)$ of some covariates $x_{i}$ represented by a parameter vector α, and the regression functions $f_{i}\left(\theta_{k}\right)$ are Gaussian regressors $\phi (\cdot ,\mu \left(x_{i};\beta_{k}\right),\sigma_{k}^{2})$ with semi-parametric (non-)linear mean functions $\mu (x_{i};\beta_{k})$. This flexible modeling allows us to better capture more non-linear relationships in the functional data via the semi-parametric mean functions. Heterogeneity is accommodated via the mixture distribution, and spatial organization can be captured via spatial-dependent mixture weights.

### 3.2. Spatial Mixture of Functional Regressions for Dual-Energy CT Images

We propose a spatialized mixture of functional regressions model, adapted to the given type of image data, for the model-based clustering of dual-energy CT scans. The images we analyze include spectral curves for each 3D voxel. Each image, denoted Im, is represented as a sample of *n* observations, Im={vi,xi,yi}i=1n where $v_{i}=\left(v_{i1},v_{i2},v_{i3}\right)$ is the *i*th voxel 3D spatial coordinates.The *i*th voxel is represented by the curve $\left(x_{i},y_{i}\right)$ composed of HU attenuation values $y_{i}=\left(y_{i1},\ldots ,y_{im}\right)$ measured at energy levels (covariates) $x_{i}=\left(x_{i1},\ldots ,x_{im}\right)$, with *m* being the number of energy levels.

To accommodate the spatial organization of the image together with the functional nature of each of its voxels, we propose spatialized conditional extensions of the general family of model (1), in which we model the *i*th voxel observation of the image using the conditional density $f(y_{i}x_{i},v_{i};\theta )$ that relates the attenuation curve levels $y_{i}$, given the associated energy levels $x_{i}$, and spatial location $v_{i}$ via a convex combination of (non-)linear (semi-)parametric functional regressors $f(y_{i}x_{i};\theta_{k})$ with spatial weights $\pi_{k}\left(v_{i};\alpha \right)$, that is,

(5) $$f\left(y_{i}x_{i},v_{i};\theta \right)=\sum_{k=1}^{K}\pi_{k}\left(v_{i};\alpha \right)f\left(y_{i}x_{i};\theta_{k}\right)\;.$$

To this purpose, we consider two different spatial constructions of the mixing weights (gating functions) $\pi_{k}\left(v_{i};\alpha \right)$: (i) softmax gates; and (ii) normalized Gaussian gates. The latter is an appropriate choice if more approximation quality is needed, and facilitates the computations in the learning process. We also consider different families to model the functional regressors, including spline and B-spline regression functions that enjoy better curve approximation capabilities, compared to linear or polynomial regression functions.

#### 3.2.1. Functional Regression Components

We have a 3D image volume over a range of energy levels that provide, for each voxel *i*, an attenuation curve $\left(x_{i},y_{i}\right)$ which represents energy-dependent changes in attenuation, which enables a better characterization of the tissue at voxel *i*. We therefore model the component densities $f(y_{i}x_{i};\theta_{k})$ as functional regression models constructed upon the attenuation curves as functional observations. This allows us to accommodate the spectral curve nature of the data. More specifically, in the case of univariate energy levels, we use smooth functional approximations to model, for the *i*th voxel, the mean spectral curve of the *k*th component $\mu \left(x_{i};\beta_{k}\right)=E_{\theta }\left[Y_{i}Z_{i}=k,x_{i}\right]$, that is, $\mu \left(x_{i};\beta_{k}\right)=\left(\mu \left(x_{i1};\beta_{k}\right),\ldots ,\mu \left(x_{im};\beta_{k}\right)\right)$ using polynomial or (B)-spline functions, whose coefficients are $\beta_{k}$.

The conditional density model for each regression is then modeled as a functional Gaussian regressor defined by $f\left(y_{i}x_{i};\theta_{k}\right)=\phi_{m}\left(y_{i};\mu \left(x_{i};\beta_{k}\right),\sigma_{k}^{2}I\right)$, with $\mu \left(x_{i};\beta_{k}\right)=B\left(x_{i}\right)\beta_{k}$ being the function approximation onto polynomial or (B-)spline bases $B(x_{i})$, and the matrix form of the functional regression model is then given by

(6) $$f\left(y_{i}x_{i};\theta_{k}\right)=\phi_{m}\left(y_{i};B\left(x_{i}\right)\beta_{k},\sigma_{k}^{2}I\right)\;,$$

where $\theta_{k}={\left(\beta_{k}^{⊤},\sigma_{k}^{2}\right)}^{⊤}\in R^{p+q+2}$ is the unknown parameter vector of regression *k*.

#### 3.2.2. Spatial Gating Functions

The constructed functional mixture of regressions model (5) specifically integrates the spatial constraints in the mixture weights $\pi_{k}\left(v_{i};\alpha \right)$ via functions of the spatial locations $v_{i}$ parametrized by vectors of coefficients α. We investigate two choices to this end. The first proposed model is a spatial softmax-gated functional mixture of regression and is defined by (5) with a softmax gating function:

(7) $$\pi_{k}\left(v_{i};\alpha \right)=\frac{\exp\left(\alpha_{k}^{⊤}v_{i}\right)}{1+\sum_{k^{\prime }=1}^{K-1}\exp\left(\alpha_{k^{\prime }}^{⊤}v_{i}\right)},$$

where $\alpha ={\left(\alpha_{1}^{⊤},\ldots ,\alpha_{K}^{⊤}\right)}^{⊤}$ is the unknown parameter vector of the gating functions. We will refer to this model, defined by (5)–(7), as the spatial softmax-gated mixture of functional regressions, abbreviated as **SsMFR**. The softmax modeling of the mixture weights is a standard choice known in the mixtures-of-experts community. However, its optimization performed at the M step of the EM algorithm, is not analytic and requires numerical Newton–Raphson optimization. This can become costly, especially in larger image applications, such as the one we address.

In the second proposed model, we use a spatial Gaussian-gated functional mixture of regressions, defined by (5) with a Gaussian-gated function:

(8) $$\pi_{k}\left(v_{i};\alpha \right)=\frac{w_{k}\phi_{3}\left(v_{i};\mu_{k},R_{k}\right)}{\sum_{\ell =1}^{K}w_{\ell }\phi_{3}\left(v_{i};\mu_{\ell },R_{\ell }\right)}\;,$$

in which $w_{k}$ are non-negative weights that sum to one, $\phi_{d}\left(v_{i};\mu_{k},R_{k}\right)$ is the density function of a multivariate Gaussian vector of dimension *d* with mean $\mu_{k}$ and covariance matrix $\Sigma_{k}$, and $\alpha ={\left(\alpha_{1}^{⊤},\ldots ,\alpha_{K}^{⊤}\right)}^{⊤}$ is the parameter vector of the gating functions with $\alpha_{k}={\left(w_{k},\mu_{k}^{⊤},\mathrm{vech}{\left(R_{k}\right)}^{⊤}\right)}^{⊤}$.

We will refer to this model, defined by (5), (6) and (8), as the spatial Gaussian-gated mixture of functional regressions, abbreviated as **SgMFR**. This Gaussian gating function was introduced in [42] to bypass the need for an additional numerical optimization in the inner loop of the EM algorithm. We obtain a closed form updating formula at the M-Step, that is detailed in the next section presenting the derived EM algorithm.

#### 3.2.3. MLE of the SgMFR Model via the EM Algorithm

Based on Equations (5), (6) and (8), the SgMFR joint density $f(y_{i},x_{i},v_{i};\theta )$ is then derived and the joint log-likelihood we maximize via EM is

(9) $$\log L\left(\theta \right)=\sum_{i=1}^{n}\log f\left(y_{i},x_{i},v_{i};\theta \right)=\sum_{i=1}^{n}\log\sum_{k=1}^{K}w_{k}\phi_{3}\left(v_{i};\mu_{k},R_{k}\right)\phi_{m}\left(y_{i};B\left(x_{i}\right)\beta_{k},\sigma_{k}^{2}I\right)\cdot$$

The complete-data log-likelihood, upon which the EM algorithm is constructed, is

(10) $$\log L_{c}\left(\theta \right)=\sum_{i=1}^{n}\sum_{k=1}^{K}Z_{ik}\log\left[w_{k}\phi_{3}\left(v_{i};\mu_{k},R_{k}\right)\phi_{m}\left(y_{i};B\left(x_{i}\right)\beta_{k},\sigma_{k}^{2}I\right)\right],$$

where $Z_{ik}$ is an indicator variable such that $Z_{ik}=1$ if $Z_{i}=k$ (i.e., if the *i*th pair $\left(x_{i},y_{i}\right)$ is generated from the *k*th regression component) and $Z_{ik}=0$, otherwise. The EM algorithm, after starting with an initial solution $\theta^{\left(0\right)}$, alternates between the E and M steps until convergence (when there is no longer a significant change in the log-likelihood).

**The E-step**: Compute the conditional expectation of the complete-data log-likelihood (10), given the image Imn and the current estimate $\theta^{\left(t\right)}$:

(11) Q(θ;θ(t))=ELc(θ)|Imn;θ(t)=∑i=1n∑k=1Kτik(t)logαkϕ3(vi;μk,Rk)ϕm(yi;B(xi)βk,σk2I),

where $\tau_{ik}^{\left(t\right)}=P\left(Z_{i}=ky_{i},x_{i},v_{i};\theta^{\left(t\right)}\right)$ given by

(12) $$\tau_{ik}^{\left(t\right)}=\frac{w_{k}^{\left(t\right)}\phi_{3}\left(v_{i};\mu_{k}^{\left(t\right)},R_{k}^{\left(t\right)}\right)\phi_{m}\left(y_{i};B\left(x_{i}\right)\beta_{k}^{\left(t\right)},{\sigma_{k}^{2}}^{\left(t\right)}I\right)}{f(v_{i},x_{i},y_{i};\theta^{\left(t\right)})}$$

is the posterior probability that the observed pair $\left(x_{i},y_{i}\right)$ is generated by the *k*th regressor. This step therefore only requires the computation of the posterior component membership probabilities $\tau_{ik}^{\left(t\right)}$(i=1,…,n), for k=1,…,K.

**The M-step**: Calculate the parameter vector update $\theta^{\left(t+1\right)}$ by maximizing the *Q*-function (11), i.e., $\theta^{\left(t+1\right)}=\arg\max_{\theta }Q\left(\theta ;\theta^{\left(t\right)}\right)$. By decomposing the Q−function as

(13) $$Q\left(\theta ;\theta^{\left(t\right)}\right)=\sum_{k=1}^{K}Q\left(\alpha_{k};\theta^{\left(t\right)}\right)+Q\left(\theta_{k};\theta^{\left(t\right)}\right),$$

with $Q\left(\alpha_{k};\theta^{\left(t\right)}\right)=\sum_{i=1}^{n}\tau_{ik}^{\left(t\right)}\log\left[w_{k}\phi_{3}\left(v_{i};\mu_{k},R_{k}\right)\right]$ and $Q\left(\theta_{k};\theta^{\left(t\right)}\right)=\sum_{i=1}^{n}\tau_{ik}^{\left(t\right)}\log\left[\phi_{m}\left(y_{i};B\left(x_{i}\right)\beta_{k},\sigma_{k}^{2}I\right)\right]$, the maximization can then be performed by *K* separate maximizations with respect to the parameters of the gating and the regression functions.

*Updating the gating functions parameters:* Maximizing (13) with respect to $\alpha_{k}$’s corresponds to the M step of a Gaussian mixture model [32]. The closed-form expressions for updating the parameters are given by

(14) $$\begin{array}{cc} \;\;\;\;w_{k}^{\left(t+1\right)} & =\sum_{i=1}^{n}\tau_{ik}^{\left(t\right)}/n, \end{array}$$

(15) $$\begin{array}{cc} \;\;\;\;\mu_{k}^{\left(t+1\right)} & =\sum_{i=1}^{n}\tau_{ik}^{\left(t\right)}v_{i}/\sum_{i=1}^{n}\tau_{ik}^{\left(t\right)}, \end{array}$$

(16) $$\begin{array}{cc} \;\;\;\;R_{k}^{\left(t+1\right)} & =\sum_{i=1}^{n}\tau_{ik}^{\left(t\right)}\left(v_{i}-\mu_{k}^{\left(t+1\right)}\right){\left(v_{i}-\mu_{k}^{\left(t+1\right)}\right)}^{⊤}\;\;/\;\sum_{i=1}^{n}\tau_{ik}^{\left(t\right)}\cdot \end{array}$$

*Updating the regression functions parameters:* Maximizing (13) with respect to $\theta_{k}$ corresponds to the M step of standard mixtures of experts with univariate Gaussian regressions. The closed-form updating formulas are given by

(17) $$\begin{array}{cc} \; & \beta_{k}^{\left(t+1\right)}={\left[\sum_{i=1}^{n}\tau_{ik}^{\left(t\right)}B^{⊤}\left(x_{i}\right)B\left(x_{i}\right)\right]}^{-1}\sum_{i=1}^{n}\tau_{ik}^{\left(t\right)}B{\left(x_{i}\right)}^{⊤}y_{i}\;, \end{array}$$

(18) $$\begin{array}{cc} \; & {\sigma_{k}^{2}}^{\left(t+1\right)}=\sum_{i=1}^{n}\tau_{ik}^{\left(t\right)}{\left(y_{i}-B\left(x_{i}\right)\beta_{k}^{\left(t+1\right)}\right)}^{2}/\sum_{i=1}^{n}\tau_{ik}^{\left(t\right)}m_{i}\;\cdot \end{array}$$

### 3.3. Alternative Two-Fold Approaches

We also investigate an alternative approach to the one derived before, which consists of a two-fold approach, rather than a simultaneous functional approximation and model estimation for segmentation. We first construct approximations of the functional data onto polynomial or (B-)splines bases $B(x_{i})$ via MLE (ordinary least squares in this case) to obtain

(19) $${\hat{\beta }}_{i}={\left[B{\left(x_{i}\right)}^{⊤}B\left(x_{i}\right)\right]}^{-1}B{\left(x_{i}\right)}^{⊤}y_{i}.$$

Then, we model the density of the resulting coefficient vectors ${\hat{\beta }}_{i}$, which is regarded as the *i*th curve representative, by a mixture density with spatial weights of the form

(20) $$f\left({\hat{\beta }}_{i},v_{i},\theta \right)=\sum_{k=1}^{K}\pi_{k}\left(v_{i};\alpha \right)\phi_{d}\left({\hat{\beta }}_{i};m_{k},C_{k}\right)\;,$$

where $m_{k}$ and $C_{k}$ are the mean and the covariance matrix of each component. The spatial weights $\pi_{k}\left(v_{i};\alpha \right)$ are normalized Gaussians as in (8) or softmax as in (7). We will refer to these methods as spatial Gaussian-gated (resp. softmax-gated) mixtures of vectorized functional regressions, **SgMVFR** (resp. **SsMVFR**).

The EM algorithm for fitting this mixture of spatial mixtures, constructed upon pre-computed polynomial or (B-)spline coefficients with its two variants for modeling the spatial weights, takes a similar form to the previously presented algorithm, and is summarized as follows. The conditional memberships of the E step are given for the softmax-gated model by

(21) $$\tau_{ik}^{\left(t\right)}=\frac{\pi_{k}\left(v_{i};\alpha^{\left(t\right)}\right)\phi_{d}\left({\hat{\beta }}_{i};m_{k}^{\left(t\right)},C_{k}^{\left(t\right)}\right)}{f({\hat{\beta }}_{i}v_{i};\theta^{\left(t\right)})}\;,$$

and for the Gaussian-gated model by

(22) $$\begin{array}{cc} \tau_{ik}^{\left(t\right)}\; & =\frac{w_{k}^{\left(t\right)}\phi_{3}\left(v_{i};\mu_{k}^{\left(t\right)},R_{k}^{\left(t\right)}\right)\phi_{d}\left({\hat{\beta }}_{i};m_{k}^{\left(t\right)},C_{k}^{\left(t\right)}\right)}{f(v_{i},{\hat{\beta }}_{i};\theta^{\left(t\right)})}\;. \end{array}$$

The latter has the same advantage as explained above. In the M step, the gating functions parameter updates are given by (14)–(16) for the Gaussian-gated model, or through a Newton–Raphson optimization algorithm for the softmax-gated model. The component parameter updates are those of classical multivariate Gaussian mixtures

(23) $$\begin{array}{cc} m_{k}^{\left(t+1\right)} & =\sum_{i=1}^{n}\tau_{ik}^{\left(t\right)}{\hat{\beta }}_{i}/\;\sum_{i=1}^{n}\tau_{ik}^{\left(t\right)}, \end{array}$$

(24) $$\begin{array}{cc} C_{k}^{\left(t+1\right)} & =\sum_{i=1}^{n}\tau_{ik}^{\left(t\right)}\left({\hat{\beta }}_{i}-m_{k}^{\left(t+1\right)}\right){\left({\hat{\beta }}_{i}-m_{k}^{\left(t+1\right)}\right)}^{⊤}\;/\;\sum_{i=1}^{n}\tau_{ik}^{\left(t\right)}\cdot \end{array}$$

In a nutshell, to compute a clustering of the image, the label of voxel *i*, given the fitted parameters $\hat{\theta }$, is calculated by the Bayes’ allocation rule (3), in which Im(i) is the spatial coordinates of voxel *i* with either its direct spectral curve representative $\left(x_{i},y_{i}\right)$ or its pre-calculated functional approximation coefficients ${\hat{\beta }}_{i}$ given by (19).

Appendix A contains the pseudo-codes summarizing the proposed method.

### 3.4. Time Complexity of the Proposed Algorithms

In this subsection we investigate the time complexity of the proposed algorithms. The time complexity of the E-step of the proposed EM algorithms for the SgMFR and the SsMFR models is of $O(Kd^{2}pnm)$, with *n* being the number of voxels, *m* the number of energy levels, *d* is the number of spatial coordinates (2 or 3), *p* the number of the number of regression coefficients, and *K* the number of clusters. For the M step, the SgMFR and the SgMVFR have closed-form updates; The SgMFR requires the calculation of the regression coefficients via weighted least squares with a complexity of $O(Kp^{2}nm)$. The SgMVFR models require the calculations of the Gaussian means and covariance matrices as in multivariate Gaussian mixtures, and have a complexity of $O(Kp^{2}n)$. However, the SsMFR and SsMVFR algorithms require at each iteration inside the M step of the EM algorithm an IRLS loop and the inversion of the Hessian matrix which is of dimension d(K−1). Therefore, the complexity of the IRLS is approximately of $O(I_{IRLS}d^{2}K^{2})$, where IRLS is the average number of iterations required by the internal IRLS algorithm. The complexity here can therefore be an issue for a large number of clusters, and the SgMFR and SgMVFR algorithms can be preferred.

## 4. Experimental Study

In this section, we describe the evaluation of different versions of our method: mixtures of B-spline and polynomial functional regressions with spatial Gaussian gates (resp. SgMFR-Bspl and SgMFR-poly), mixture of B-spline regressions with softmax gates (SsMFR-Bspl), and mixture of vectorized B-spline regressions with Gaussian gates (SgMVFR-Bspl).

### 4.1. Data

In total, 91 head and neck DECT scans were evaluated, consisting of HNSCC tumors of different sizes and stages from different primary sites. In our dataset, 34% of tumors are located in the oral cavity, 26% in the oropharynx, 21% in the larynx, 8% in the hypopharynx and 11% in other locations. The tumors’ T-stage [43] ranges from T1 to T4. Of the patients, 75% were coming for a first diagnostic while 25% were recurrent patients. Institutional review board approval was obtained for this study with a waiver of informed consent. Tumors were contoured by an expert head and neck radiologist. All scans were acquired using a fast kVp switching DECT scanner (GE Healthcare) after administration of IV contrast and reconstructed into 1.25 mm sections of axial slices with a resolution of 0.61 mm, as previously described [11]. Multienergy VMIs were reconstructed at energy levels from 40 to 140 keV in 5 keV increments at the GE Advantage workstation (4.6; GE Healthcare).

In each DECT scan, we crop volumes of interest (VOIs) of size 150*150*6 containing a tumor, along with the 21-point-spectral curve associated to each selected voxel, in order to reduce the computational demands for an exploratory study, and to exclude regions containing a majority of air voxels around the body. A pre-processing step is also applied to mask any remaining air voxels in the VOI to focus the clustering on tissues.

### 4.2. Regularization Parameter

In our study, we augmented the statistical estimator in Equation (16) of the covariance matrix of the spatial coordinates within cluster *k*, with a regularization parameter λ∈(0,1], which controls the amount of spatial dispersion (neighborhood) taken into account in the spatial mixture weights, by

(25) $${\tilde{R}}_{k}^{\left(t+1\right)}=\lambda R_{k}^{\left(t+1\right)}.$$

By doing so, we can numerically control the amount of data within cluster *k* (i.e., its volume). Indeed, if we decompose $C_{k}=\lambda DAD^{T}$ where A is the $C_{k}$ eigenvectors matrix, and D is a diagonal matrix whose diagonal elements are the eigenvalues in decreasing order, then λ is the volume of cluster *k*. Since the tumor cluster has in general no strong spatial dispersion, then in practice, we take small values of order 0.1.

### 4.3. Parameter Initialization

For the sake of reproducibility, we start by initializing the regression mixture and weight parameters of the EM algorithm with a coarse clustering solution given by a Voronoi diagram. We build up Voronoi tiles over the selected voxels in the VOI (a square region where air voxels are deleted) with the k-means algorithm applied only on spatial coordinates. Then to fix the number of clusters *K*, and to fix the spatio-spectral hyper-parameter λ, we assess on a small training set (10 patients) the three metrics described in Section 4.5. In our experiments, *K* is taken to be large enough, say 20, 30 or 40, so that we do not have to perform a full grid search which could be computationally demanding, and the value λ around 0.075 works very well. The process is run similarly for both methods of mixtures of functional regressions and mixtures of vectorized functional regressions. The range of search values is adapted for each method, and 5 search values are taken in each range. In the end, we use the mean of optimal values over the patients in the train set.

### 4.4. Baselines

We compare the quantitative and qualitative performance of our methodology with three baseline algorithms. First, we implement a Gaussian mixture model (GMM) with the iterative EM algorithm, using the standard non-reshaped algorithm [32] to cluster the spectral curves, thus leading to not include spatial coordinates. Because several clusters can become empty through the optimization, we fix an initial number of clusters (K=150) that ends up providing, on average, the same resulting number of clusters for our method (i.e., K=40). Second, we implement k-means clustering, using all vector information available, that is, the input vector is built with spectral information (i.e., the energy decay curve points) concatenated to a vector of spatial information (i.e., the 3D coordinates). The number of clusters is picked to be also K=40, the number of clusters being stable throughout the optimization. We use the Matlab k-means implementation for images with a reproducible initialization through the built-in ‘imsegkmeans’ function. Third and last, we implement selective search, a machine learning graph-based segmentation method for object recognition. Using a region merging hierarchical approach with an SVM classifier to select the hierarchical rank of the resulting regions, the authors published an open-source code [44]. We apply selective search on low-, intermediate- and high-energy levels (resp. 40, 65 and 140 keV). These energy levels are used instead of the three RGB image channels. We note that selective search does not predetermine the number of clusters, but specifies a cluster minimum size or favors smaller or larger cluster sizes.

### 4.5. Metrics

Our clustering methods, as well as the baseline clustering algorithms, are all evaluated using the following three metrics:

1. A cluster separation index, *Davies–Bouldin index (DB),* computed on spatial content and on spectral content.
2. A clustering separation index focused only on the relationship between tumor clusters and other clusters, *Davies–Bouldin index on tumor (DB${}_{t}$),* an adaptation of DB, computed on spatial and on spectral content.
3. A segmentation score computed on tumor clusters versus ground truth region, *the Dice similarity score,* that can be computed only on spatial content.

To define tumor clusters, we select the cluster(s) which best cover the tumor, i.e., the ones that give the best Dice score when merged together. Several clusters segmenting the tumor area can indeed represent different tumor subparts, but we only know the tumor primary site contour as the ground truth.

When *C* is the ensemble of clusters, d(·,·) is the Euclidean distance operator, $\bar{c_{k}}$ is the centroid of cluster $c_{k}$, the Davies–Bouldin index is defined as

(26) $$\;\;\;\;\mathrm{DB}\left(C\right)\;=\;1/C\sum_{c_{k}\in C}\max_{c_{l}\in C\backslash c_{k}}\left(S\left(c_{k}\right)+S\left(c_{l}\right)\right)/d\left(\bar{c_{k}},\bar{c_{l}}\right)$$

where $S\left(c_{k}\right)=1/c_{k}\sum_{x_{i}\in c_{k}}d\left(x_{i},\bar{c_{k}}\right).$ The ‘tumor’ Davies–Bouldin index is adapted as

(27) $$\;\;\;{\mathrm{DB}}_{t}\left(C\right)\;=\;\max_{c_{l}\in C\backslash c_{tum}}\left(S\left(c_{tum}\right)+S\left(c_{l}\right)\right)/d\left(\bar{c_{tum}},\bar{c_{l}}\right)),$$

where $c_{tum}$ is the region of the merged tumor clusters. The Dice score is defined as

(28) $$\mathrm{Dice}\left(c_{tum},c_{truth}\right)=2*c_{tum}\cap c_{truth}/\left(c_{tum}+c_{truth}\right)\;,$$

where $c_{truth}$ is the ground truth region. We summarize the distribution of these index values across the population by computing the mean, median and interquartile range.

## 5. Results

**Figure 2:**
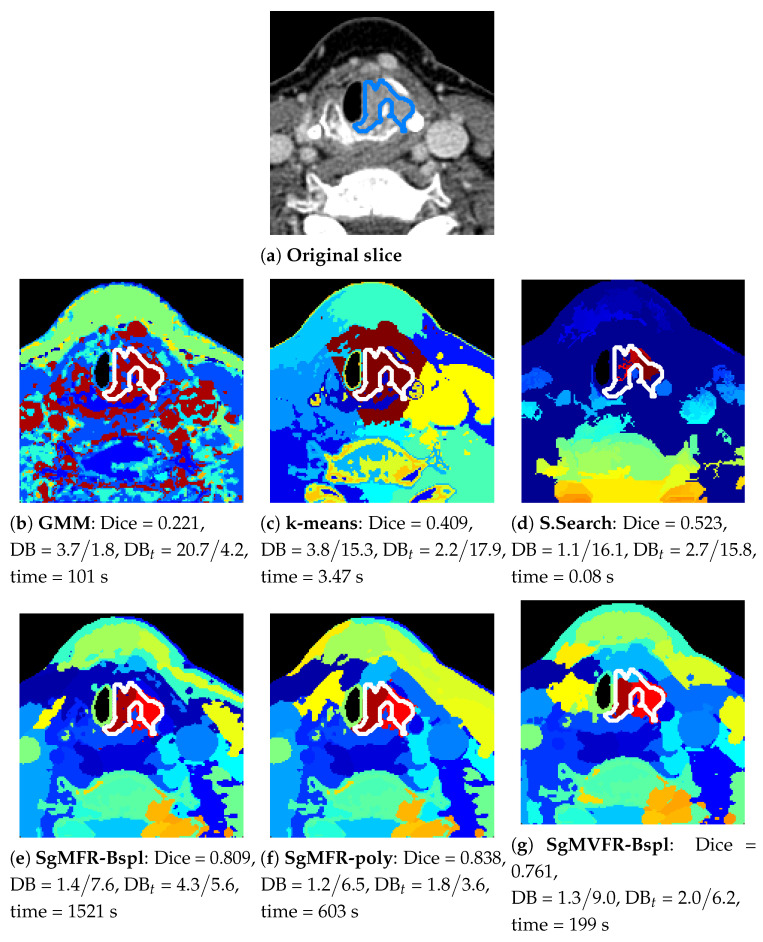
Clustering results for each approach in one tumor (DB${}_{\left(t\right)}=$ spatial/spectral index). Our proposed approaches are on the bottom row. One random color is assigned per cluster, ground truth tumor contour is in blue (**a**) or white (**b**–**g**).

Figure 2 shows an overview of our results for one tumor example. We visualize the results on a 2D slice when the model has been run on the 3D VOI containing this slice.

The top row shows the performance of the baseline algorithms, whereas the bottom row shows our proposed methods. While the baseline approaches attribute a high number of clusters to bone regions containing big spectral variations and miss smaller variations in tissue regions, our methods with Gaussian gates in Figure 2e–g are able to adapt to relative variations and split the image with more spatial coherence. The results also demonstrate that our method is able to capture tissue characteristics invisible in Figure 2b–d,h. Note that DB and DB${}_{t}$ scores depend on the number of clusters and SsMFR in Figure 2h has a very low number of clusters (softmax having vanishing clusters in the optimization). GMM and selective search in Figure 2b,d have around 40 clusters (varying number as explained in Section 4.4). The results in Figure 2c,e–g were obtained with 40 clusters.

**Figure 3:**
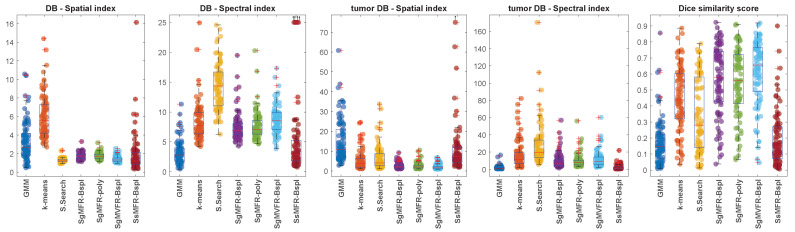
Boxplots for each metric per method. Note that SsMFR-Bspl method gives few outliers out of reach (order of $10^{13}$) on the DB spectral index, and one outlier of 135 for tumor DB spatial index. These values are shifted in the displayed range and exhibit a top arrow.

Table 1 and Figure 3 present the quantitative results obtained with the three clustering metrics defined in Section 4.5. Among the three proposed methods that outperform the baseline methods in terms of Dice score (SgMFR-Bspl, SgMFR-poly, and SgMVFR-Bspl), we compared the Dice score distribution obtained with SgMFR-poly (which has the lowest median Dice score among the three) to that obtained with the k-means-like baseline (which has the highest median Dice score) with a two-sample *t*-test, obtaining *p* = 0.0014.

**Figure 4:**
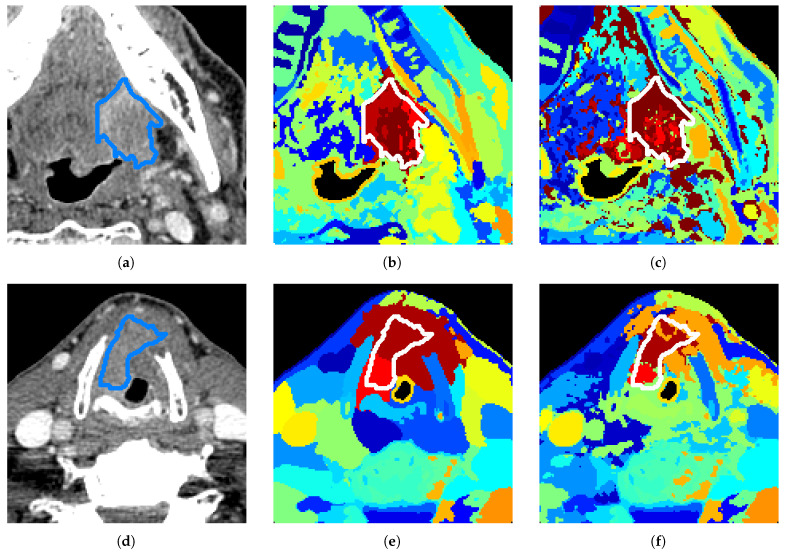
Clustering results for our SgMFR method with different λ tuning. (**a**) Original slice. (**b**) λ=0.075, Dice = 0.79, DB = 1.68/7.29, DB${}_{t}$ = 1.47/6.79. (**c**) λ=0.100, Dice = 0.39, DB = 2.59/4.27, DB${}_{t}$ = 3.34/3.74. (**d**) Original slice. (**e**) λ=0.075, Dice = 0.36, DB = 1.75/6.70, DB${}_{t}$ = 1.28/23.71. (**f**) λ=0.080, Dice = 0.64, DB = 1.87/6.57, DB${}_{t}$ = 1.78/2.93. One random color is assigned per cluster, ground truth tumor contour is in blue (**a**,**d**) or white (**b**,**c**,**e**,**f**).

**Figure 5:**
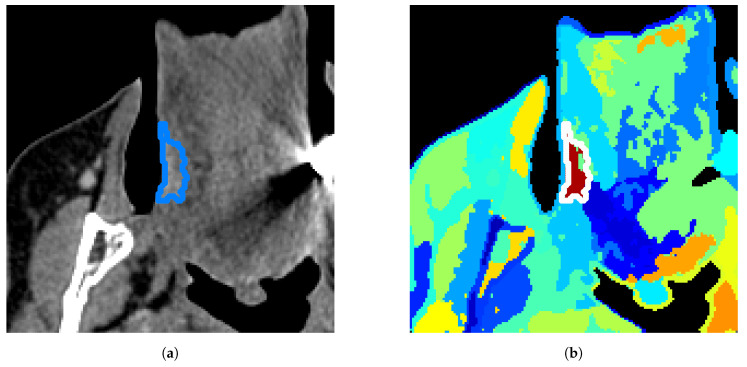
Clustering results with our SgMFR for a small tumor. Note the robustness of the result in the presence of a metallic artifact in the right-hand side of the anatomical image. (**a**) Original slice. (**b**) Dice = 0.84, DB = 1.64/6.92, DB${}_{t}$ = 1.98/7.22, λ=0.075.

Figure 4 showcases the clustering results obtained when varying the tuning of λ. Here, we understand that a smaller λ gives a higher preference to the spatial information: clusters are compact and define well-separated areas. On the other hand, a larger λ gives a higher priority to the spectral information: clusters more closely match tissue characteristics, but one cluster can be split into tiny voxel groups spread all over the image. The ideal λ choice would be a λ that prioritizes spectral information, but still achieves some cluster spatial compactness. We assess this through metrics calculated on spectral and spatial content as explained in Section 4.5. The general tuning of λ=0.075 is determined to be, on average, the optimal hyper-parameter. However, we can see strong improvements in tumor separation, on a case by case basis, with small variation of λ. As shown in Figure 4e,f, the Dice score increases from 0.36 to 0.64. This shows an example out of several results belonging to the lower quartile in the boxplot of Dice scores in Figure 3 that could be highly improved simply with a specific tuning. Some other examples of results in the lower quartile could be due to small tumors (size inferior to 1cm), although half of these small tumors are actually well-separated with our method, reaching a Dice score as high as 0.84 in the best case (see in Figure 5).

The proposed algorithms clearly outperform the investigated standard clustering algorithms in terms of the considered metrics, such as the Dice score. In particular, the two approaches based on Gaussian-gating mixture weights, whenever they are directly built upon a functional mixture model (SgMFR), or used with a prior functional data representation of the energy curves (SgMVFR), enjoy both high segmentation capabilities while being computationally effective. The two proposed alternative approaches based on the use of softmax-gating mixture weights (SsMFR and SsMVFR), can, however, be computationally expensive, given that they use, at each EM iteration, a Newton–Raphson optimization; they may lead in some situations to less precise segmentation, typically due to a numerical convergence issue, as compared to the proposed Gaussian-gating-based approaches. As a result, we can suggest to the interested users prioritize the use of the SgMFR and SgMVF approaches when investigating our proposed techniques for DECT clustering. This being said, in order to investigate the statistical significance of the differences in the results of the proposed family of algorithms, it is interesting to perform a statistical study with appropriate statistical testing.

## 6. Discussion and Conclusions

In this paper, we developed a statistical methodology to cluster intensity attenuation curves in DECT scans. We applied our proposed methods, together with other alternative clustering algorithms used as baselines, to a set of 91 DECT scans of HNSCC tumors. The classical manner of evaluating algorithms for clustering/segmentation is via measures of overlap (such as the Dice score) with a ground truth segmentation. However, as mentioned in the Introduction above, the manual segmentations of HNSCC tumors that are used as “ground truth” can suffer from large inter-rater variability, and do not incorporate in any systematic manner regions immediately adjacent to the tumor that may be biologically important for determining the course of evolution of the tumor. Because of this inherent uncertainty in the appropriate contours of an HNSCC tumor, the main objective in our paper was to compare our clustering results to the manual contouring, but also to explore associations between voxels within the ground truth tumor contour and voxels in the surrounding tissue areas.

Compared to the baseline algorithms, it is clear both visually and quantitatively that our methods using Gaussian gates (SgM(V)FR) produce results that match better the manual segmentation contours. Our method using softmax gates (SsMFR) is less flexible compared to the one with Gaussian gating functions, and thus sometimes leads to non-satisfactory results. Although in terms of qualitative assessment, clusters of SsMFR-Bspl are indeed more spatially compact, quantitative performance in some situations stays similar to GMM baseline. Thus, this variant of the algorithm does not appear to perform well in practice. Using Gaussian gates, however, Dice score distributions are significantly better than the k-means-like algorithm, the best of our baseline methods.

That being said, it is also clear that with Dice scores ranging from nearly 0 to nearly 1, our proposed methods do not recover the “ground truth” segmentations in a reliable and consistent manner. Several reasons may explain this finding. First, our clinical dataset of DECT scans is not uniform, i.e., it includes tumors of highly variable characteristics, in highly variable sizes, locations and environments, which makes it particularly challenging. Moreover, as seen in Figure 4, changes in parameter tuning can lead to substantial improvement in Dice scores for some tumors. Finally, because of their intricate morphology and often small sizes, HNSCC tumors are inherently difficult to segment. In a recent international challenge, Dice scores of head and neck tumor segmentation ranged across different competition entries between 0.56 and 0.76 [45].

Most importantly, as argued throughout this paper, the clinical value of recovering the manual segmentations of HNSCC tumors as an objective criterion for evaluating the algorithm is also not clear. In fact, it was recently argued in the clinical literature that AI methods in medical imaging would be more meaningful if evaluated against clinical outcomes, as opposed to an evaluation against radiologists’ performance, due to inherent subjectivity and variability of the latter [46]. For all the reasons, the objective of this paper was moved away from reproducing the manual contours produced by the radiologist, and was focused instead on developing tools that discover patterns of association in the DECT data.

Our study has several limitations. As discussed above, in our view, the appropriate way of evaluating the methodology’s clinical utility is not by computing Dice scores relative to manually drawn contours. Rather, a more clinically informative evaluation would determine the performance of the recovered clusters in predicting clinical outcome in a machine learning setting, compared to the same predictive algorithm applied with the manual tumor segmentations. Such an evaluation is missing from the present paper; it will be part of a subsequent paper in future work. Another limitation stems from the lack of an automated identification of those clusters that are associated with the tumor region. Right now, we choose those clusters that maximize overlap with the manually segmented tumor region. Ideally, however, the abnormal tumor clusters should be identified automatically, by selecting those clusters that have the highest association with clinical outcomes. In this manner, the automated cluster identification can be naturally made part of a single machine learning pipeline for predicting clinical outcomes. Yet another limitation comes from the very small size of the subset of tumors (*n* = 10) over which we estimated the algorithm parameters (λ and number of clusters), before applying the algorithm to the remaining 81 tumors in our dataset. A larger dataset, together with additional patient-specific tuning will help tune the algorithm’s performance.

The need for the improvements described above is clear, and they will be made part of a subsequent publication. In the present article, we chose to focus on the theoretical and algorithmic developments. As mentioned in the Introduction, this is the first time to our knowledge that statistical tools from the functional data analysis field are put into practice with DECT data. As such, the present paper remains an inherently exploratory one in its experimental framework.

Nevertheless, we believe that we provide several important technical and methodological contributions. We constructed a functional regression mixture model that integrates spatial content into the mixture weights, and we developed a dedicated EM algorithm to estimate the optimal model parameters. Our mixture-based model is a highly flexible statistical approach allowing for many choices of the parametric form of the component densities. We proposed two candidate designs for the mixture weights, normalized Gaussian gates and softmax gates. The Gaussian-gate closed-form solution for spatial mixture weight updates considerably reduces the computation time while also providing solutions with better clustering index values, compared to the Newton–Raphson optimization algorithm needed at each update of the softmax-gating parameters.

## Figures and Tables

**Table 1:** Average median (interquartile range) of metrics and runtime over 81 patient scans. DB cluster separation index is computed for spatial content (spat-DB) and spectral content (spec-DB); idem for DB${}_{t}$ indices focused on tumor separability; runtime is given in seconds.

GMM				k-Means			S.Search			SgMFR-Bspl			SgMFR-poly			SgMVFR-Bspl			SsMFR-Bspl		
	**avg**	**med**	**itq**	**avg**	**med**	**itq**	**avg**	**med**	**itq**	**avg**	**med**	**itq**	**avg**	**med**	**itq**	**avg**	**med**	**itq**	**avg**	**med**	**itq**
Dice	0.17	0.15	*(0.15)*	0.45	0.44	*(0.28)*	0.35	0.28	*(0.44)*	0.56	0.58	*(0.32)*	0.57	0.56	*(0.31)*	0.61	0.66	*(0.28)*	0.20	0.15	*(0.20)*
spat-DB	3.32	2.69	*(2.38)*	6.00	5.57	*(3.08)*	1.30	1.28	*(0.24)*	1.75	1.75	*(0.45)*	1.74	1.72	*(0.45)*	1.40	1.32	*(0.35)*	1.84	1.22	*(1.31)*
spec-DB	3.17	2.80	*(2.21)*	8.41	7.75	*(3.51)*	14.36	14.36	*(5.66)*	7.52	6.95	*(1.83)*	7.66	7.13	*(2.27)*	8.69	8.58	*(2.81)*	3.78	2.96	*(3.48)*
spat-DB${}_{t}$	14.97	10.95	*(13.94)*	5.07	3.68	*(4.30)*	6.57	4.07	*(6.00)*	2.49	2.05	*(1.27)*	2.59	2.00	*(1.33)*	2.05	1.70	*(0.95)*	11.07	7.12	*(7.89)*
spec-DB${}_{t}$	1.97	1.11	*(1.71)*	16.52	11.42	*(12.17)*	28.47	19.86	*(20.11)*	9.64	6.78	*(6.90)*	9.46	7.52	*(5.46)*	11.57	9.53	*(8.64)*	2.52	1.36	*(2.20)*
runtime	342	165	*(210)*	3.64	3.78	*(0.78)*	0.062	0.047	*(0.014)*	1423	1321	*(600)*	902	901	*(345)*	437	361	*(277)*	2425	2121	*(2049)*

## Data Availability

The source codes for the proposed methods are publicly available at https://github.com/fchamroukhi/DECT-CLUST (accessed on 28 October 2022), free of charge.

## Abbreviations

The following abbreviations are used in this manuscript:

DECT	Dual-energy computed tomography
DSC	Dice similarity score
EM	Expectation–maximization
FDA	Functional data analysis
fPCA	Functional PCA
HNSCC	Head and neck squamous cell carcinoma
MLE	Maximum likelihood estimation
PCA	Principal component analysis
R-CNN	Regional convolutional neural network
SgMFR	Spatial Gaussian-gated mixtures of functional regressions
SgMVFR	Spatial Gaussian-gated mixtures of vectorized functional regressions
SsMFR	Spatial softmax-gated mixtures of functional regressions
SsMVFR	Spatial softmax-gated mixtures of vectorized functional regressions
VOI	Volumes of interest
VMI	Virtual monochromatic image

## Appendix A. Pseudo-Codes for the Proposed Methods

**Algorithm A1** Pseudo code of DECT-CLUST with SsMFR and SgMFR models.
**Inputs:** 4D-image (*n* curves $(v_{i},x_{i},y_{i}{)}_{i=1}^{n}$, # clusters *K*, degree *p* (and # knots *q*) 1: Initialization:$\theta^{\left(0\right)}=\left(\alpha^{\left(0\right)},\theta_{1}^{\left(0\right)},\ldots ,\theta_{K}^{\left(0\right)}\right)$; set t←0 2: **while** increment in log-likelihood >ϵ (e.g., $1e^{-6}$) **do** 3: E-Step: % Conditional memberships : 4: **for** k=1,…,K **do** 5: compute $\tau_{ik}^{\left(t\right)}$ for i=1,…,n using (12) for SgMFR or the standard one for SsMFR 6: **end for** 7: M-Step: %a. Spatial Mixture Weights 8: **if** SgMFR model is used: **then** 9: %Update Spatial Gaussian-Gating Functions: 10: **for** k=1,…,K **do** 11: compute $w_{k}^{\left(t+1\right)}$ (14), $\mu_{k}^{\left(t+1\right)}$ (15), and $R_{k}^{\left(t+1\right)}$ (16) 12: **end for** 13: **end if** 14: **if** SsMFR model is used: **then** 15: % Update Spatial Softmax-Gating Functions: 16: *IRLS Algorithm*: 17: **Initialize:** $\alpha^{\left(s\right)}\leftarrow \alpha^{\left(t\right)}$ and s←0 (IRLS iteration) 18: **while** increment in $Q_{\alpha }\left(\alpha ,\theta^{\left(t\right)}\right)>\delta$ (eg. 1e-6) **do** 19: compute $\alpha^{\left(s+1\right)}$ using *IRLS* 20: s←s+1 21: **end while** 22: $\alpha^{\left(t+1\right)}\leftarrow \alpha^{\left(s\right)}$ 23: **end if** 24: %b. Update Functional Mixture Components: 25: **for** k=1,…,K **do** 26: compute $\beta_{k}^{\left(t+1\right)}$ using (17) and $\sigma_{k}^{2(t+1)}$ using (18) 27: **end for** 28: % Convergence test 29: Compute the joint log-likelihood (9) for SgMFR or the standard marginal log-likelihood for SsMFR. 30: t←t+1 31: **end while** **Outputs:**$\hat{\theta }=\left(\alpha^{\left(t\right)},\theta_{1}^{\left(t\right)},\ldots \theta_{K}^{\left(t\right)}\right)$ the MLE of θ and the conditional probabilities $\tau_{ik}^{\left(t\right)}$

**Algorithm A2** Pseudo code of DECT-CLUST with alternative SgMVFR and SsMVFR models.
**Inputs:** 4D-image (*n* curves $(v_{i},x_{i},y_{i}{)}_{i=1}^{n}$, # clusters *K*, degree *p* (and # knots *q*) 1: **for** i=1,…,n **do** 2: Compute the functional data representations $\hat{\beta_{i}}$ by (19) 3: **end for** 4: Initialization:$\theta^{\left(0\right)}=\left(\alpha^{\left(0\right)},\theta_{1}^{\left(0\right)},\ldots ,\theta_{K}^{\left(0\right)}\right)$; set t←0 (EM iteration) 5: **while** increment in log-likelihood >ϵ (eg. 1e-6) **do** 6: E-Step: % Conditional memberships : 7: **for** k=1,…,K **do** 8: compute $\tau_{ik}^{\left(t\right)}$ for i=1,…,n using (21) for SsMVFR or using (22) for SgMVFR 9: **end for** 10: M-Step: %(a) Update Spatial Weights: 11: **if** SgMVFR model is used: **then** 12: %Update Spatial Gaussian-Gating Functions: 13: **for** k=1,…,K **do** 14: compute $w_{k}^{\left(t+1\right)}$ using (14), $\mu_{k}^{\left(t+1\right)}$ using (15), and $R_{k}^{\left(t+1\right)}$ using (16) 15: **end for** 16: **end if** 17: **if** SsMVFR model is used: **then** 18: % Update Spatial Softmax-Gating Functions: 19: $\alpha^{\left(t+1\right)}\leftarrow$ is returned by *IRLS*: 20: **end if** 21: %(b) Update Multivariate Mixture Components: 22: **for** k=1,…,K **do** 23: compute $m_{k}^{\left(t+1\right)}$ using (23) and $C_{k}^{\left(t+1\right)}$ using (24) 24: **end for** 25: % Convergence test 26: Compute the joint log-likelihood (9) for SsMVFR or the standard marginal one for SgMVFR. 27: t←t+1 28: **end while** **Outputs:**$\hat{\theta }=\left(\alpha^{\left(t\right)},\theta_{1}^{\left(t\right)},\ldots \theta_{K}^{\left(t\right)}\right)$ the MLE of θ and the conditional probabilities $\tau_{ik}^{\left(t\right)}$
